# Anti-SARS-CoV-2 B and T-Cell Immune Responses Persist 12 Months After mRNA Vaccination with BNT162b2 in Systemic Lupus Erythematosus Patients Independently of Immunosuppressive Therapies

**DOI:** 10.3390/vaccines13040396

**Published:** 2025-04-09

**Authors:** Mario Ferraioli, Alessandra Aiello, Immacolata Prevete, Maria Sole Chimenti, Luigi De Marco, Silvia Meschi, Davide Mariotti, Valentina Vanini, Gilda Cuzzi, Andrea Salmi, Stefania Notari, Valeria Mellini, Vincenzo Puro, Fabrizio Maggi, Delia Goletti, Gian Domenico Sebastiani

**Affiliations:** 1 Rheumatology, Immunology and Clinical Allergology Unit, Department of Systems Medicine, University of Rome Tor Vergata, 00133 Rome, Italy; maria.sole.chimenti@uniroma2.it (M.S.C.); luigi.demarco12@gmail.com (L.D.M.); 2 Translational Research Unit, National Institute for Infectious Diseases Lazzaro Spallanzani-IRCCS, 00149 Rome, Italy; alessandra.aiello@inmi.it (A.A.); valentina.vanini@inmi.it (V.V.); gilda.cuzzi@inmi.it (G.C.); andrea.salmi@inmi.it (A.S.); valeria.mellini@inmi.it (V.M.); 3 U.O.C. Reumatologia, Azienda Ospedaliera San Camillo-Forlanini, 00152 Rome, Italy; iprevete@scamilloforlanini.rm.it (I.P.); gsebastiani@scamilloforlanini.rm.it (G.D.S.); 4 Laboratory of Virology, National Institute for Infectious Diseases Lazzaro Spallanzani-IRCCS, 00149 Rome, Italy; silvia.meschi@inmi.it (S.M.); davide.mariotti@inmi.it (D.M.); fabrizio.maggi@inmi.it (F.M.); 5 UOS Professioni Sanitarie Tecniche, National Institute for Infectious Diseases Lazzaro Spallanzani-IRCCS, 00149 Rome, Italy; 6 Cellular Immunology and Pharmacology Laboratory, National Institute for Infectious Diseases Lazzaro Spallanzani-IRCCS, 00149 Rome, Italy; stefania.notari@inmi.it; 7 U.O.C. Risk Management, National Institute for Infectious Diseases Lazzaro Spallanzani-IRCCS, 00149 Rome, Italy; enzopuro3113@gmail.com

**Keywords:** mRNA vaccine, vaccination, SLE, therapy, immune response, COVID-19

## Abstract

**Background:** In response to the SARS-CoV-2 pandemic, a massive vaccination campaign was launched. Nonetheless, concerns arose regarding some peculiar groups of patients, including those affected by Systemic Lupus Erythematosus (SLE), because of the immune-suppressive drugs routinely administered to patients and the risk of possible disease flares. Since the effects of the third booster vaccination in SLE have been poorly assessed, this study aims to evaluate the immunogenicity and safety of the third BNT162b2 vaccine dose, together with the effects of immunosuppressive drugs. **Methods:** A monocentric SLE cohort and a cohort of age- and sex-matched healthy controls (HCs) (all vaccinated with three homologous doses) were consecutively enrolled 6 months (T1) after their third vaccine shot. Vaccine immunogenicity was evaluated by analyzing humoral and cellular immune responses at T1 and 12 months (T2). Vaccine safety was evaluated by assessing adverse events related to vaccination (T0) and comparing disease activity among T0, T1, and T2. Effects of immunosuppressive drugs were assessed by stratifying patients according to therapy at vaccination: (1) receiving (IS) or (2) not receiving immunosuppressive drugs (Non-IS). **Results:** At T1, the humoral responses were comparable between SLE and HC subjects, while the cellular response was significantly higher in HC (*p* = 0.01). No differences were found at T2 between cohorts. Similarly, both at T1 and T2, the immune responses of IS and Non-IS groups were comparable. Moreover, lupus disease flares were limited and mostly mild, and no life-threatening adverse events were reported. **Conclusions:** The booster BNT162b2 vaccine is safe and induces an immune response, which is persistent and not affected by ongoing immunosuppressive drugs.

## 1. Introduction

The SARS-CoV-2 pandemic has been one of the most serious and disruptive diseases of the last 20 years. The COronaVIrus Disease 2019 (COVID-19), which may follow SARS-CoV-2 infection, has been responsible for more than seven million deaths since its outbreak [1]. In response, a worldwide vaccination campaign was launched, and some groups of patients considered at a higher risk of severe COVID-19 outcomes were prioritized, including those affected by Systemic Lupus Erythematosus (SLE) [2,3,4]. Indeed, patients affected by autoimmune diseases are known to have a higher risk of infections [5,6] and complications from COVID-19 than the general population [7,8,9,10]. It has been reported that SLE patients are at a higher risk of SARS-CoV-2 infection and worse outcomes from COVID-19 [11,12,13,14,15]. This is likely due to SLE’s intrinsic immune dysfunction and the administrated medications [16,17,18]. Drugs routinely used to treat SLE are immunomodulators and immunosuppressants and have been associated with an increased risk of death from COVID-19 [19]. In addition, phase III trials on different vaccines (including mRNA-1273, BNT162b2 mRNA, and Ad26.COV2) did not include patients treated with immunosuppressants or immune-modifying drugs within six months of enrolment [20]. Thus, at the start of the vaccination campaign, there was a complete lack of efficacy and safety data in patients with autoimmune rheumatological diseases [21,22,23,24,25].

Various studies have explored the SARS-CoV-2 vaccines’ immunogenicity in SLE and other autoimmune rheumatic diseases [26,27,28,29,30,31]. Factors affecting the anti-SARS-CoV-2 response have been investigated, despite limitations. Namely, data were reported after only one vaccine dose or in grouped patients with multiple rheumatic diseases or vaccinated with different vaccines or heterologous schedules. A meta-analysis conducted in 2021 showed that SLE patients had a lower seroconversion rate compared to both Rheumatoid Arthritis (RA) patients and healthy controls (HCs) [20]. Subsequent literature reviews with meta-analyses assessed the vaccine’s immunogenicity [27,32]. The authors presented satisfactory data on the humoral response, although limited information was available concerning patients following booster vaccinations [33]. Similarly, out of a few studies that evaluated the cellular response in SLE patients, none encompassed subjects after the third dose, and only in one cohort were all patients vaccinated with mRNA vaccines [34]. The T-cell response has been shown to play an important role in SARS-CoV-2 infection [26,35,36,37] in both immune-competent and vulnerable individuals, such as those with multiple sclerosis [38,39,40,41], rheumatological diseases [42], or transplants [43,44,45]. Interestingly, T-cell responses are stable and effective against all variants in healthy subjects and immune-suppressed subjects [26,46].

Given the evidence, this study aimed to examine the immune response and safety of administering three doses of the BNT162b2 vaccine in a real-world population with SLE. Additionally, it sought to assess the impact of immunosuppressive therapies and other potential influencing factors.

## 2. Materials and Methods

### 2.1. Study Cohort and Design

This a prospective, real-life study investigating the immune efficacy and safety of the third dose of mRNA vaccine BNT162b2 (Pfizer–BioNTech) in a monocentric cohort of consecutively enrolled SLE patients.

Patients diagnosed with SLE according to the 2019 European League Against Rheumatism/American College of Rheumatology (EULAR/ACR) criteria [47] were consecutively enrolled at the Rheumatology Unit of San Camillo-Forlanini Hospital (Rome, Italy) between March 2022 and February 2023.

SLE patients were enrolled 6 months after their third vaccination shot (T1), and the following data were collected: demographic, clinical [including disease activity and SLE-related autoantibodies (Ab)], and prior SARS-CoV-2 infection. Moreover, vaccine-related adverse events (AEs), clinical data, previous SARS-CoV-2 infection, and ongoing therapies were also retrospectively collected regarding the time of their third vaccination (T0). A follow-up evaluation was performed 6 months after inclusion (T2; i.e., 12 months after the third dose) collecting clinical data and possibly SARS-CoV-2 infection if it had occurred.

SLE patients who were receiving care at the lupus outpatient clinic of the Rheumatology Unit of San Camillo-Forlanini Hospital were invited to participate in this study if they met the following inclusion criteria: (1) age > 18 and <70 years, (2) SLE diagnosis before the SARS-CoV-2 outbreak (February 2020), (3) vaccination with 3 doses of the BNT162b2 mRNA vaccine, and (4) absence of SARS-CoV-2 infection.

Exclusion criteria included (1) high disease activity (SLEDAI > 10) or an ongoing disease flare [based on SELENA-SLEDAI flare index (SSFI)] at inclusion [48], (2) pregnancy or breast-feeding during the study period, (3) any diagnosis of cancer in the last 5 years, (4) liver disease (Child-Pugg ≥ B), (5) acute or chronic kidney injury (eGFR < 30 mL/min), and (6) treatment with rituximab in the last 12 months or intravenous immunoglobulins in the last 2 months. Notably, all patients who contracted SARS-CoV-2 infection (i.e., positive to the antigenic/molecular test on the swab sample and/or positive for anti-nucleoprotein immunoglobulin G serology) during the study period were excluded from the analysis.

Disease activity was assessed using the Safety of Estrogens in Lupus Erythematosus National Assessment version of the Systemic Lupus Erythematosus Disease Activity Index (SELENA-SLEDAI), together with the “Physician Global Assessment Index” (PGA). The SSFI was used to assess disease flares [48]. SLE-related Ab included anti-nuclear antibodies (ANAs) and anti-double-strand DNA antibodies (anti-dsDNA). Moreover, both SLE-related Ab and disease activity indexes were registered at all time points. Lastly, the following laboratory items were collected related to T0 only: leukocyte count, C3, C4, erythrocyte sedimentation rate (ESR), and C-reactive protein (CRP). The impact of the following parameters (collected at T0) on both humoral and cellular immune responses was analyzed: age, sex, BMI, ANA, anti-dsDNA, PGA, SELENA-SLEDAI, lymphocyte count, C3, C4, ESR, and CRP. They were selected for their relevance to immune function, immune activation, and disease activity in SLE.

Ongoing therapies at vaccination (T0) encompassed hydroxychloroquine, mycophenolate mofetil, azathioprine, belimumab, methotrexate, cyclosporine A, corticosteroids, and their prednisone equivalent dosage.

According to the ongoing therapies at T0, SLE patients were divided into two groups:

- IS: including those who were receiving an immune-suppressor drug at the time of vaccination, regardless of hydroxychloroquine, and/or any corticosteroids at a dosage higher than 5 mg prednisone equivalent per day;
- Non-IS: including only patients who were not receiving any immune-suppressor drug at the time of vaccination [e.g., hydroxychloroquine or corticosteroids alone (at a dosage lower or equal to 5 mg prednisone equivalent per day) or their eventual combination or patients not receiving any therapies].

As the healthy control group, a convenience sample of healthcare workers (HCs), matched on age and sex, was enrolled 6 months after their third BNT162b2 mRNA vaccine shot at INMI Lazzaro Spallanzani. The same inclusion and exclusion criteria (when applicable) were used. Like SLE patients, demographic data were collected at T1 and blood samples at T1 and T2.

### 2.2. Humoral and Cellular Immune Responses

To assess humoral and cellular immune responses to the BNT162b2 mRNA vaccine, blood samples were collected at T1 and T2 in lithium heparinized tubes (BD Vacutainer, Becton Dickinson, Florence, Italy, Cat. 367526) at the Rheumatology Unit of San Camillo-Forlanini Hospital and processed at INMI within 2 h of collection.

Humoral and cellular immune responses were assessed by analyzing specific antibodies (Ab) and interferon (IFN)-γ-specific responses, respectively. Serology was assessed by quantifying neutralizing antibodies (Neu Abs) through a microneutralization assay, using hCoV-19/Italy/LAZ-INMI-3329/2022 (Accession ID EPI_ISL_13300234 Omicron Lineage BA.5.1) and anti-receptor-binding domain (RBD) IgG (anti-RBD Abs) (Architect^®^ i2000sr Abbott Diagnostics, Chicago, IL, USA). The specific T-cell response was evaluated using a whole-blood platform. Plasma IFN-γ levels released after overnight stimulation with SARS-CoV-2 Spike protein peptides (PepTivator^®^ Prot_S1, Prot_S, and Prot_S+, Miltenyi Biotec, Bergisch Gladbach, Germany, Cat. 130-127-048, Cat. 130-126-701 and Cat. 130-127-312, respectively), and with staphylococcal enterotoxin B (SEB) (Merck Life Science, Milan, Italy, Cat. S4881) as a positive control, were measured using an ELLA Simple Plex Human IFN-gamma (3rd Gen.) Assay (Bio-Techne, Minneapolis, MN, USA, Cat. SPCKB-PS-002574), as previously described [49,50,51]. The positive control was used to verify the competence of the immune systems of the enrolled subjects. All subjects responded to SEB stimulus and were included in the analysis. The following cut-offs were used to define a humoral and cellular immune response as positive: anti-RBD Abs ≥ 7.1 BAU/mL; neutralizing Abs > 8 reciprocals of dilution and spike ≥ 16 pg/mL.

### 2.3. Statistical Analyses

Data were analyzed using GraphPad software version 9.3.1 (GraphPad Prism, San Diego, CA, USA). Categorical variables were reported as frequencies (percentages). Quantitative measures were reported as means ± standard deviations in case of a normal distribution, or medians (interquartile range) otherwise. The normality of distribution was evaluated using the D’Agostino normality test. Pairwise comparisons of groups were conducted using the Mann–Whitney test, Wilcoxon Signed-Rank test, and Student’s *t* test, when appropriate; the Kruskal–Wallis test was used for comparisons among groups followed by Dunn’s multiple comparison test. Chi-square and Fisher tests and McNemar’s test were used for proportions (unpaired and paired data, respectively). Correlations were evaluated by a nonparametric Spearman’s rank test and reported with the rho coefficient. A *p* value < 0.05 was considered significant.

### 2.4. Ethics Statement

This research was approved by the local Ethical Committee of “Lazio 1” from the San Camillo-Forlanini Hospital (Rome, Italy), approval number 149/CE Lazio 1, and by the Ethical Committee of the National Institute of Infectious Diseases (INMI) “L. Spallanzani” IRCCS (approval numbers 297/2021, 247/2021, and 319/2021) for the enrollment of healthy controls. All subjects provided written informed consent.

## 3. Results

### 3.1. Characteristics of the Enrolled Population

Out of 51 SLE patients screened for this study [52], 15 (29.4%) were excluded from analyses due to SARS-CoV-2 infection occurring between T0 and T1 (Table 1). Therefore, 36 SLE patients and 43 HCs were included; none of them were smokers. Regarding therapies, no patients had discontinued medication at the time of their third dose. In particular, at vaccination, 25 (69.4%) patients were receiving hydroxychloroquine, 9 (25%) mycophenolate mofetil, 4 (11.1%) azathioprine, 3 (8.3%) belimumab, and 1 (2.7%) methotrexate and cyclosporine A, respectively. Corticosteroids were administrated to 14 (38.8%) patients, 1 (2.7%) at a dosage > 5 mg prednisone equivalent, while all the others were at a dosage ≤ 5 mg prednisone equivalent; the median corticosteroid dosage was 2.5 (0–5) mg prednisone equivalent. On the other hand, five (13.8%) SLE patients were not in therapy. Consequently, 16 (44.4%) patients were included in the IS group and 20 (55.6%) in the Non-IS group.

### 3.2. Humoral and Cellular Response

**Figure 1:**
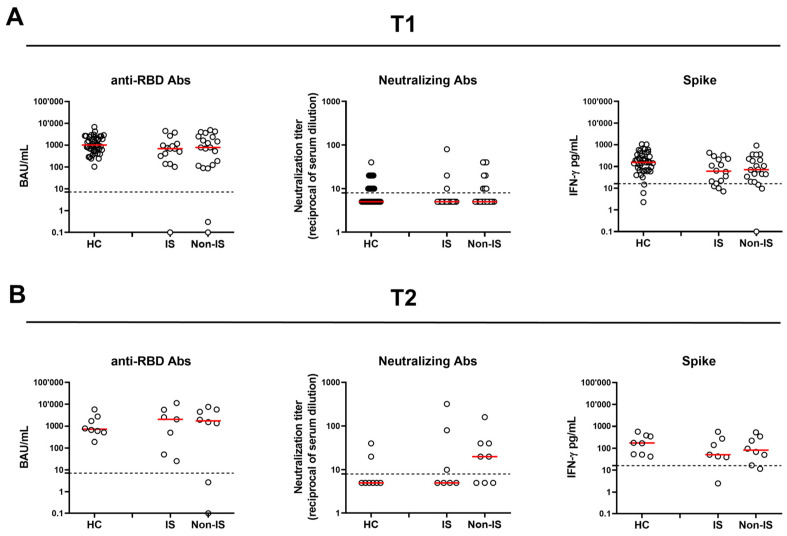
Humoral and spike-specific IFN-γ T-cell response in SLE patients and HCs at 6 months (T1) (**A**) and 12 months (T2) (**B**) after the third vaccine dose. SLE patients were stratified according to the therapy in two groups: IS and Non-IS. Anti-RBD Abs were reported as binding antibody units (BAU)/mL, while neutralizing antibodies were reported as reciprocals of serum dilution, respectively. Spike-specific IFN-γ levels were subtracted from the unstimulated control value. Red horizontal lines indicate medians. The cut-off of each test is indicated by dashed lines. For the statistical analysis, the Kruskal–Wallis test followed by Dunn’s multiple comparison test was used. If not reported, *p* values are to be considered non-significant. HC: healthy control; RBD: receptor-binding domain; IS: immune-suppressed patients; Non-IS: non-immune-suppressed patients.

At T1, a comparable qualitative and quantitative response was observed when comparing SLE patients with HCs for both anti-RBD Abs and Neu Abs. Similarly, no significant differences were found when comparing IS and Non-IS groups and when comparing those with HCs. On the other hand, the HC cohort showed a significantly higher spike-induced IFN-γ level [154.4 (67.3–345)] than SLE patients [66.7 (20.1–219.4)] (*p* = 0.01), while no differences were found between the two therapy groups (Table 2 and Figure 1A).

At T2, out of 19 (52.7%) SLE patients who returned to follow-up, 4 (11.1%) had contracted SARS-CoV-2 after T1; consequently, only 15 (41.6%) SLE patients were included in the analyses. As for HCs, eight (18.6%) did not have SARS-CoV-2 infection and were available (Table 3, Figure 1B). No significant differences were found between SLE patients and HCs and between therapy groups regarding the antibody or cellular response, either qualitatively or quantitatively.

**Figure 2:**
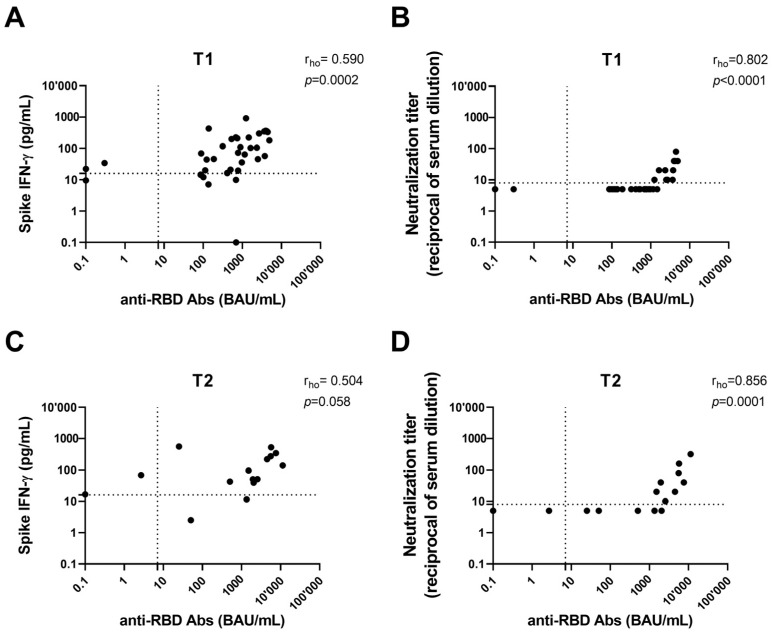
Correlation between antibody response (anti-RBD and neutralizing antibodies) and both spike-specific IFN-γ levels and neutralization titer after 6 months (T1) (**A**,**B**) and 12 months (T2) from the third dose (**C**,**D**). Anti-RBD and neutralizing antibodies were indicated as binding antibody units (BAU)/mL and reciprocals of serum dilution, respectively. IFN-γ levels were subtracted from the unstimulated control value. The cut-off of each test is indicated by dashed lines. IFN: interferon; RBD: receptor-binding domain.

A significant correlation was found at 6 months between anti-RBD Abs and Neu Abs and the T-cell response (*p* < 0.0001 and *p* = 0.0002, respectively) (Figure 2A,B). The correlation between anti-RBDs and Neu Abs was still observed at 12 months despite fewer samples (*p* = 0.0001), while for anti-RBD Abs and the T-cell response, there was no correlation (Figure 2C,D). The data presented do not allow us to conclude whether the effect diminishes over time or if the reduction is due to the smaller sample size at 12 months.

#### 3.2.1. Immune Response’s Longitudinal Observation

**Figure 3:**
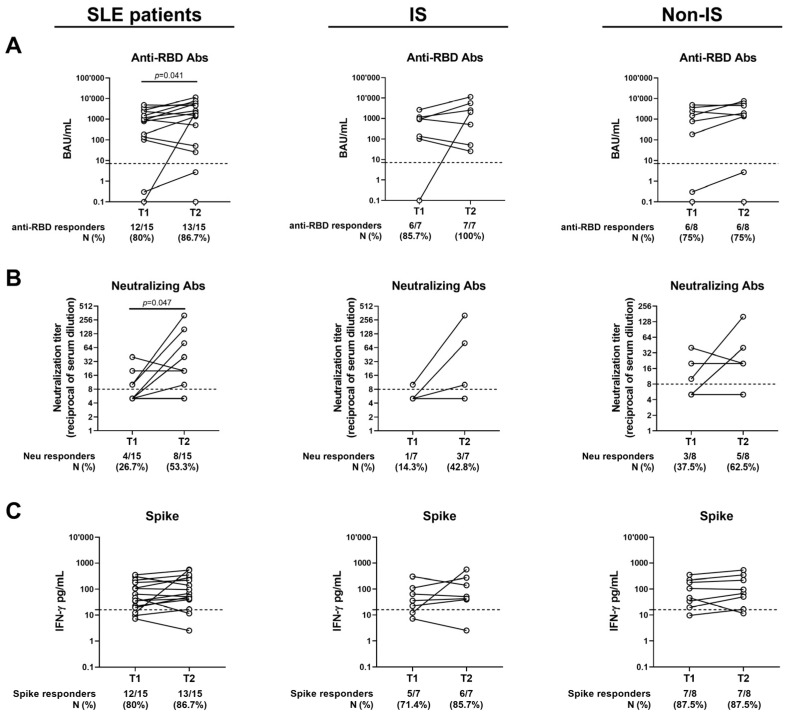
Kinetics of the humoral and T-cell responses after 6 months (T1) and 12 months (T2) from the third vaccine dose. Only patients who completed the follow-up were included. (**A**) Anti-RBD Abs expressed as BAU/mL; (**B**) neutralizing Abs expressed as reciprocal of serum dilution; (**C**) spike-specific IFN-γ levels expressed as pg/mL. The cut-off of each test is indicated by dashed lines. Wilcoxon Signed-Rank test was used. If not reported, *p* values are to be considered non-significant. M: median; N: number; IQR: interquartile range; Ab: antibodies; RBD: receptor-binding domain; IS: immune-suppressed patients; Non-IS: non-immune-suppressed patients.

The humoral and cellular responses to SARS-CoV-2 vaccination were evaluated in the SLE patients (n = 15) longitudinally sampled from T1 to T2 (Table 4 and Figure 3A–C). No significant differences were found regarding the spike-specific T-cell response, in terms of both the magnitude and proportion of responders (Figure 3C). On the other hand, both anti-RBD and Neu Abs significantly differed from T1 to T2 (Figure 3A,B). Indeed, the anti-RBD antibody titer significantly increased from 897.5 (101–2358) BAU/mL at T1 to 1958 (50.6–5611) BAU/mL at T2 (*p* = 0.04), but without a significant difference in terms of seroconversion rate. Moreover, the proportion of SLE patients showing neutralizing activity significantly increased at T2 (*p* = 0.04), as well as the magnitude of Neu Abs, which showed a 2-fold increase from T1 to T2 [T1: 5 (5–10) vs. T2: 10 (5–40), *p* = 0.04].

#### 3.2.2. Possible Factors Influencing the Immune Response

The possible association of the immune response with baseline factors was investigated by stratifying patients according to age, sex, BMI, ANA, anti-dsDNA, PGA, SELENA-SLEDAI, lymphocyte count, C3, C4, ESR, and CRP. Data from the univariate analysis showed that a younger age (lower than 50 years) was associated with a higher anti-RBD Ab titer (*p* = 0.01). Similarly, a significant negative correlation was found between CRP values and the IFN-γ-specific T-cell response (*p* = 0.02). No associations were found for any of the other parameters explored with either cellular or humoral responses (Table 5).

### 3.3. Vaccine’s Safety

To evaluate the vaccine’s safety in SLE patients, AE, disease activity items, and SLE-related Ab were analyzed and compared between the two therapy groups.

#### 3.3.1. Adverse Events

All 36 SLE patients (100%) included in this study reported at least one symptom in the 7 days following the third dose. Most of them had between two and four symptoms (47.2%). The most frequently reported one was pain at the injection site, followed by fatigue (52.7%) and arthralgia (47.2%) or fever (47.2%). No differences in AE incidence between the two groups were found (Table 6).

#### 3.3.2. Disease Activity

Overall, SLE disease activity remained stable during the observation period, and even if some flares occurred, their incidence remained low (Table 7). Indeed, no significant differences were found for SELENA-SLEDAI and PGA during the observation period between SLE patients, as well as between the IS and Non-IS groups. Most SLE flares were of mild/moderate severity, and no significant differences were found between treatment groups. Similarly, the Ab positivity rate did not change during the one-year follow-up and did not differ among groups, except for ANA. The latter increased in the SLE group between T0 and T1 (*p* = 0.03), and it decreased in the Non-IS subgroup when comparing T1 and T2 (*p* = 0.02) (Table 7). Lastly, no statistical differences were found regarding values of the lymphocyte count, C3, C4, ESR, and CRP between groups at T0.

## 4. Discussion

This study investigated the immunogenicity and safety of the third dose of the BNT162b2 vaccine on a real-life single-center cohort of SLE patients, comparing it with a sex- and age-matched cohort of HCs and exploring the potential effects of immunosuppressive drugs taken at vaccination on these outcomes.

To the best of our knowledge, this is the first study to comprehensively evaluate the immune response to the third dose of BNT162b2 in a real-life SLE cohort followed for such a long period.

Overall, the findings from this study show both the safety and immunogenicity of the third anti-SARS-CoV-2 mRNA vaccination dose in SLE patients, enriching current knowledge on the topic.

First, we showed that SARS-CoV-2 vaccination successfully induced in SLE patients both humoral and cellular immunity, which persisted over the 12-month-long follow-up. SLE and HC differed only in the magnitude of the cellular response assessed 6 months after the third dose, with a lower response observed in SLE patients compared to the HC cohort. Notably, IS drugs did not appear to affect the immune response, as no significant differences in terms of the humoral and cellular response were found between the two therapy groups, as well as when compared to HCs. Therefore, it is possible to argue that the efficacy of the BNT162b2 mRNA booster vaccine in SLE patients is comparable to that observed in HCs.

The immunogenicity of the third BNT162b2 mRNA dose was also confirmed by the longitudinal analysis. SLE patients who completed the 1-year follow-up showed an overall increasing trend in both components of immunity, particularly the humoral response, which showed a significant 2-fold increase. This finding is particularly interesting as no other study in the literature has reported a similar increase in healthy controls [53] or immune-suppressed subjects [54]. However, we cannot rule out the possibility that the increased humoral response may be due to cases of asymptomatic SARS-CoV-2 infection not detected through a positive swab or anti-N-IgG serology test.

Evidence from the literature is only partially comparable since most studies assessed immunogenicity following two vaccine doses and no longer than 6 months after vaccination. Indeed, a recent meta-analysis retrieved, for the humoral response following mRNA vaccines, a pooled seropositive rate of 91.3% (95% CI: 83.0–97.3%) [27]. Moreover, SLE patients evaluated after a booster BNT162b2 dose in two different cohorts reported data comparable with those outlined in the present study for both anti-RBD Ab [33,55] and neutralizing Ab [33]. Particularly, Larsen et al. found no differences in antibody production between immune-suppressed (with high-dose prednisolone and DMARDs) and non-immune-suppressed patients [55]. Additionally, Sartori et al. observed elevated seropositivity rates for anti-SARS-CoV-2 in SLE patients, who received a complete three-dose vaccination schedule. They also noted that immune-suppressed SLE patients did not exhibit a diminished response [56].

As for the cellular response, the presented data appear higher—as expected—than those reported after two doses, which range from 45% to 63% [27]. On the other hand, after a BNT162b2 booster dose, Schiavoni et al. detected an IFN-γ T-cell specific response in 60% of their SLE patients [57], while Assawasaksakul et al. found it in 94% of them, even though the latter were previously vaccinated with inactivated vaccines [33].

Lastly, among the possible associated factors analyzed in this study, only two were found to be related to the immune response: younger age was associated with a higher anti-RBD antibody response, while an inverse association was found between CRP levels and the cellular response. Consistent with other studies, no associations were found with gender, disease activity, anti-dsDNA, IS therapies, C3 and C4 levels, and lymphocyte count [27,31,34]. At the same, older age was found to be associated with lower Ab concentrations in a large study on HCs receiving a BNT162b2 vaccine booster dose [53], thus confirming that senescence negatively affects the immune response against infections and vaccinations [58].

As for vaccine safety, AEs and disease activity were evaluated. Most importantly, no serious AEs or life-threatening events were registered after vaccination and in the follow-up period. Almost 50% of patients reported two to seven symptoms in the week following injection, with pain at the injection site, fatigue, and arthralgia being the most reported ones. IS drugs did not influence the incidence of AE, and no relevant differences were found between the IS and Non-IS groups. Our findings are challenging to compare with the existing literature, as few studies have analyzed cohorts consisting solely of SLE patients who received three homologous doses of the BNT162b2 vaccine. Tunitsky-Lifshitz et al. reported a lower incidence of adverse events in a cohort of 17 SLE patients vaccinated with three doses of the BNT162b2 vaccine while under belimumab treatment [59]. Arthralgia or fatigue was reported in only 23% of patients, while fever was reported in 20%. On the other hand, when comparing results presented here with those from HCs, a similar incidence of fatigue and myalgia was observed, while a higher incidence of injection site pain, headache, and fever was reported in SLE patients [60]. Additionally, a recent review highlighted that the incidence of AEs in SLE patients does not differ after the first and second doses, occurring in 44.8% and 50.8% after the first and second doses, respectively [27].

The second indicator of safety was disease activity, which was evaluated by measuring SLE-related Ab. Overall, throughout the entire observation period, disease activity remained globally stable, with no differences in PGA, SELENA-SLEDAI, and the incidence of flares, along the follow-up and comparisons between therapy groups. Even in this case, our results are not easily comparable to previous studies, as they did not assess patients after three vaccine doses. Nevertheless, different authors reported the incidence of flares after two mRNA vaccine shots, and their findings are consistent with the data presented here. The incidence of flares in mRNA-vaccinated patients is reported as between 3% [61,62] and 20% [63]—with an average of 5.5% [27]—and severe ones from 0% [61] to 2% [27,31]. A significant difference lies in the evaluation period; whereas literature data were collected between 7 and 90 days post-vaccination, participants in the current study were evaluated at 6 and 12 months following the third dose.

As for the SLE-specific Abs, while anti-dsDNA Abs positivity did not increase following vaccination, ANA Abs showed a significant increase in the SLE group between T0 and T1 and markedly decreased at T2. Therefore, it appears that anti-dsDNA Abs are not induced by vaccination, unlike ANA Abs. Notably, the transient increase in ANA has also been found in inflammatory arthritis patients following anti-SARS-CoV-2 vaccination [64], as well as in COVID-19 patients [65], and can be ascribed to the activation of B cells after vaccination [66]. Furthermore, as anti-dsDNA Abs are more strictly related to disease activity, these data further confirm the safety of BNT162b2 vaccination in SLE patients.

To the best of our knowledge, no other study longitudinally evaluated anti-RBD Abs and neutralizing Abs following vaccination in SLE patients. While some studies reported a decreased ANA prevalence after the second dose in a cohort of HCs vaccinated with BNT162b2 [67], Sarin et al. [68] found no new autoantibodies post-vaccination in SLE patients, while Gerosa et al. observed more flares in those with pre-existing anti-dsDNA antibodies. Additionally, a detailed T-cell-specific response has been recorded over time [69]. Notably, this study has several strengths, such as the comparison with a healthy control group and the in-depth evaluation of the humoral response, both in terms of anti-RBD and neutralizing Abs and the spike-specific T-cell response, offering detailed immune insights following vaccination.

This study has some limitations, mainly due to the limited number of patients available after 12 months. At the same time, it should be acknowledged that few other studies enrolled larger cohorts of SLE patients, with all subjects vaccinated with three homologous doses. Additionally, other limitations of the study should be mentioned: the lack of comparison of adverse events between SLE patients and HCs, and the lack of SARS-CoV-2-specific immune profile data at T0. Such data would be valuable for assessing the direct effect of the third vaccine shot on the SARS-CoV-2-specific immune response immediately after vaccination. Without these data, the observed effects could be influenced by prior vaccine doses/SARS-CoV-2 infection/contacts.

## 5. Conclusions

Vaccination against SARS-CoV-2 has emerged as the key strategy to control and prevent the spread and severe outcomes of COVID-19, particularly in vulnerable populations such as patients with SLE. This study demonstrates that administering a booster BNT162b2 mRNA vaccine is safe and well-tolerated in SLE patients, with few instances of severe disease flares and no severe adverse events. Additionally, the vaccination elicits both humoral and T-cell-specific immune responses against SARS-CoV-2, which persist for up to one year following the booster administration. Notably, immunogenicity does not appear to be affected by immunosuppressive drugs, ensuring prolonged protection for SLE patients.

## Figures and Tables

**Table 1:** Demographic data of the enrolled SLE and HC subjects.

				*p* value	
		SLE patients	HC	Within SLE cohort	SLE vs. HC *
	Total	36	43		-
**Subjects, n (%)**	IS	16 (44.4%)		0.8	-
	Non-IS	20 (55.6%)			-
	Total	32 (88.9%)	35 (81.4%)		0.3
**Females, n (%)**	IS	13 (81.2%)		0.1	0.9
	Non-IS	19 (95%)			0.1
	Total	52.9 ± 11.7	48.1 ± 10.9		0.06
**Age, years, M ± SD**	IS	49.3 ± 11.8		0.1	0.09
	Non-IS	55.4 ± 10.9			0.2
**Disease duration,** **years, M ± SD**	Total	19.1 ± 7.8			-
	IS	17.7 ± 6.5	-	0.1	-
	Non-IS	20.2 ± 8.8			-

Footnotes: All information referred to 6 months after the third vaccine dose. M: mean; n: number; SD: standard deviation; SLE: Systemic Lupus Erythematosus; IS: patients who were receiving an immune-suppressor drug at the time of vaccination. Non-IS: patients who were not receiving an immune-suppressor drug at the time of vaccination. * compared to the total HC population.

**Table 2:** Qualitative and quantitative immune responses in SLE patients and HCs at 6 months after the third vaccine shot.

			SLE	HC	*p* value	
					Within SLE cohort	SLE vs. HC *
**anti-RBD** **antibodies**	**Qualitative response,** **n (%)**	Total	33/36 (91.7)	43/43 (100)		0.09
		IS	15/16 (93.7)		>0.9	0.2
		Non-IS	18/20 (90)			0.09
	**Quantitative response,** **BAU/mL Median (IQR)**	Total	718.7 (137–2176)	1010 (529–2348)		0.1
		IS	687 (182–1107)		>0.9	0.4
		Non-IS	779 (115.4–2460)			0.9
**Neutralizing antibodies**	**Qualitative response,** **n (%)**	Total	11/36 (30.6)	17/43 (39.5)		0.4
		IS	3/16 (18.7)		0.2	0.2
		Non-IS	8/20 (40)			>0.9
	**Quantitative response,** **reciprocal of serum dilution (IQR)**	Total	5 (5–10)	5 (5–10)		0.6
		IS	5 (5–5)		0.5	0.6
		Non-IS	5 (5–17.5)			>0.9
**Spike-specific IFN-γ T-cell response**	**Qualitative response,** **n (%)**	Total	30/36 (83.3)	40/43 (93.0)		0.2
		IS	13/16 (81.2)		>0.9	0.3
		Non-IS	17/20 (85)			0.3
	**Quantitative response,** **pg/mL Median (IQR)**	Total	66.7 (20.1–219.4)	154.4 (67.3–345)		**0.01**
		IS	60.5 (17.5–221.6)		>0.9	0.09
		Non-IS	70.9 (23.4–216.5)			0.1

Footnotes: SLE: Systemic Lupus Erythematosus; HC: healthy control; n: number; IQR: interquartile range; IS: immune-suppressed patients; Non-IS: non-immune-suppressed patients; RBD: receptor-binding domain. In bold, we report the significant *p* values. * compared to the total HC population.

**Table 3:** Qualitative and quantitative immune responses in SLE patients and HCs at 12 months after the third vaccine dose.

			SLE	HC	*p* value	
					Within SLE cohort	SLE vs. HC *
**anti-RBD** **antibodies**	**Qualitative response,** **n (%)**	Total	13/15 (86.7)	8/8 (100)		0.5
		IS	7/7 (100)		0.4	>0.9
		Non-IS	6/8 (75)			0.4
	**Quantitative response,** **BAU/mL Median (IQR)**	Total	1958 (50.6–5611)	728.4 (538.7–2487)		0.7
		IS	2044 (50.6–5611)		>0.9	>0.9
		Non-IS	1739 (340.4–5444)			>0.9
**Neutralizing antibodies**	**Qualitative response,** **n (%)**	Total	8/15 (53.3)	2/8 (25)		0.3
		IS	3/7 (42.8)		0.6	0.6
		Non-IS	5/8 (62.5)			0.3
	**Quantitative response,** **reciprocal of serum dilution (IQR)**	Total	10 (5–40)	5 (5–16.2)		0.2
		IS	5 (5–80)		>0.9	>0.9
		Non-IS	20 (5–40)			0.4
**Spike-specific IFN-γ T-cell response**	**Qualitative response,** **n (%)**	Total	13/15 (86.7)	8/8 (100)		0.5
		IS	6/7 (85.7)		>0.9	0.4
		Non-IS	7/8 (87.5)			>0.9
	**Quantitative response,** **pg/mL Median (IQR)**	Total	68.1 (39.3–274.6)	173.6 (51.6–374.6)		0.2
		IS	50.9 (39.3–274.6)		>0.9	0.7
		Non-IS	82 (24.9–315.6)			>0.9

Footnotes: SLE: Systemic Lupus Erythematosus; HC: healthy control; n: number; IQR: interquartile range; IS: immune-suppressed patients; Non-IS: non-immune-suppressed patients; RBD: receptor-binding domain. * compared to the total HC population.

**Table 4:** Longitudinal observation of qualitative and quantitative humoral and cellular responses in SLE patients.

		T1 n = 15	T2 n = 15	*p* value
**Qualitative** **response**	**anti-RBD Ab responders, n (%)**	12 (80%)	13 (86.7%)	0.3
	**Neutralizing Ab responders, n (%)**	4 (26.7%)	8 (53.3%)	**0.04**
	**Cellular responders, n (%)**	12 (80%)	13 (86.7%)	0.5
**Quantitative** **response**	**anti-RBD titers, BAU/mL, Median (IQR)**	897.5 (101–2358)	1958 (50.6–5611)	**0.04**
	**Neutralizing Ab titers, Median (IQR)**	5 (5–10)	10 (5–40)	**0.04**
	**spike IFN-γ levels, pg/mL, Median (IQR)**	45.8 (19.4–180.8)	68.1 (39.3–274.6)	0.1

Footnotes: anti-RBD Abs expressed as BAU/mL; neutralizing Abs expressed as reciprocal of serum dilution; spike IFN-γ levels expressed as pg/mL. IQR: interquartile range; n: number; Ab: antibody; RBD: receptor-binding domain. In bold, we indicate the significant *p* values.

**Table 5:** Univariate analysis of factors associated with immune responses in patients with SLE.

Characteristics	n (%)	Anti-RBD Ab (BAU/mL)			Neutralizing Ab Titer			IFN-γ (pg/mL)		
		Median (IQR)	rho	*p*	Median (IQR)	rho	*p*	Median (IQR)	rho	*p*
**Age**										
<50	11 (30.6)	1152 (530–4224)		**0.01**	5 (5–40)		0.09	181 (45–335)		0.07
≥50	25 (69.4)	682 (107–1354)			5 (5–7.5)			46 (16–166)		
**Sex**										
Female	32 (88.9)	693 (126–1589)		0.28	5 (5–10)		0.7	71 (20–219)		0.7
Male	4 (11.1)	1732 (616–2631)			8 (5–10)			40 (25–237)		
**BMI**										
<25	20 (55.6)	712 (126–3318)		>0.9	5 (5–20)		0.3	104 (21–222)		0.3
≥25	16 (44.4)	736 (150–1409)			5 (5–9)			46 (16–217)		
**ANA**										
Negative	10 (27.8)	820 (169–1298)		0.8	5 (5–6.2)		0.4	159 (32–253)		0.2
Positive	26 (72.2)	693 (133–2540)			5 (5–12.5)			61 (19–185)		
**Anti-dsDNA**										
Negative	31 (86.1)	778 (188–2494)		0.1	5 (5–10)		0.6	69 (19–223)		0.9
Positive	5 (13.9)	138 (0–1428)			5 (5–12.5)			34 (22–268)		
**PGA**										
0	32 (88.9)	760 (220–2176)		0.1	5 (5–10)		0.6	71 (25–219)		0.1
>0	4 (11.1)	68 (0–2042)			5 (5–8)			16 (8–231)		
**Lymphocyte count**			−0.05	0.7		−0.05	0.7		0.11	0.5
**C3**			0.2	0.2		0.17	0.3		0.19	0.2
**C4**			0.1	0.5		−0.07	0.6		−0.12	0.4
**ESR**			−0.29	0.08		−0.27	0.1		−0.04	0.8
**CRP**			−0.27	0.1		−0.22	0.2		−0.37	**0.02**
**SELENA-SLEDAI**			−0.25	0.1		−0.05	0.7		−0.04	0.8

Footnotes: n: number; IQR: interquartile range; RBD: receptor-binding domain; BMI: body mass index; ANAs: antinuclear antibodies; anti-dsDNA: anti-double-strand DNA; PGA: physician’s global assessment; ESR: erythrocyte sedimentation rate; CRP: C-reactive protein; IFN: interferon. In bold, we indicate the significant *p* values.

**Table 6:** Adverse events were reported by SLE patients in the 7 days following the third vaccine dose.

	SLE patients n = 36	IS n = 16	Non-IS n = 20	*p* value
**0 to 1 symptom, n (%)**	8 (22.2%)	4 (25%)	4 (20%)	0.7
**2 to 4 symptoms, n (%)**	17 (47.2%)	8 (50%)	9 (45%)	0.7
**5 to 7 symptoms, n (%)**	11 (30.5%)	4 (25%)	7 (35%)	0.5
**Injection site’s pain, n (%)**	28 (77.7%)	11 (68.7%)	17 (85%)	0.2
**Fatigue, n (%)**	19 (52.7%)	8 (50%)	11 (55%)	0.7
**Arthralgia, n (%)**	17 (47.2%)	7 (43.7%)	10 (50%)	0.7
**Myalgia, n (%)**	14 (38.8%)	6 (37.5%)	8 (40%)	0.8
**Fever, n (%)**	17 (47.2%)	7 (43.7%)	10 (50%)	0.7
**Headache, n (%)**	13 (36.1%)	6 (37.5%)	7 (35%)	0.8
**Chills, n (%)**	13 (36.1%)	5 (31.2%)	8 (40%)	0.5

Footnotes: Patients were divided according to therapy at the time of vaccination. n: number; SLE: Systemic Lupus Erythematosus; IS: patients who were receiving an immune-suppressor drug at the time of vaccination. Non-IS: patients who were not receiving an immune-suppressor drug at the time of vaccination.

**Table 7:** Disease activity indexes and SLE-related antibodies were compared among groups and at different time points.

	T0				T1				T2				*p* T0 vs. T1			***p* T0 vs. T2**			***p* T1 vs. T2**		
	SLE patients	IS	Non-IS	*p*	SLE patients	IS	Non-IS	*p*	SLE patients	IS	Non-IS	*p*									
	n = 36	n = 16	n = 20		n = 36	n = 16	n = 20		n = 15	n = 7	n = 8		SLE patients	IS	Non-IS	**SLE** **patients**	**IS**	**Non-IS**	**SLE** **patients**	**IS**	**Non-IS**
**ANA, n (%)**	26 (72.2%)	10 (62.5%)	16 (80%)	0.2	33 (91.6%)	13 (81.2%)	20 (100%)	0.1	9 (46.6%)	3 (42.8%)	6 (75%)	0.7	**0.03**	0.2	0.1	0.3	0.3	0.7	**0.006**	0.06	**0.02**
**Anti-dsDNA ab, n (%)**	5 (13.8%)	3 (18,7%)	2 (10%)	0.4	5 (13.8%)	3 (18.7%)	2 (10%)	0.4	2 (13.3%)	0 (0%)	1 (12.5%)	0.9	0.9	0.9	0.9	0.4	0.2	0.8	0.4	0.8	0.8
**PGA, M (IQR)**	0 (0–0.5)	0 (0–0.0)	0 (0–0)	0.4	0 (0–1)	0 (0–0)	0 (0–0)	0.6	0 (0–0)	0 (0–2)	0 (0–2)	0.4	0.8	0.6	0.6	0.3	0.6	0.4	0.6	0.5	0.7
**SELENA-SLEDAI, M (IQR)**	1 (0–2)	2 (0–2.7)	0 (0–2)	0.06	0.5 (0–2)	1.5 (0–2)	0 (0–2)	0.7	0 (0–2)	1 (0–2)	0 (0–2)	0.6	0.8	0.4	0.3	0.9	0.2	0.7	0.7	0.5	0.7
**SLEDAI flare index**																					
Mild/moderate, n (%)	0 (0%)	0 (0%)	0 (0%)	-	2 (5.5%)	0 (%)	2 (10%)	0.1	0 (0%)	0 (0%)	0 (0%)	-	0.1	-	0.1	-	-	-	0.1	0.5	0.3
Severe, n (%)	0 (0%)	0 (0%)	0 (0%)	-	1 (2.7%)	0 (0%)	1 (5%)	0.3	1 (6.6%)	0 (0%)	1 (12.5%)	0.3	0.3	-	0.9	0.5	-	0.1	0.5	-	0.4

Footnotes: Ab: antibodies; PGA: physician’s global assessment; SLE: Systemic Lupus Erythematosus; SELENA: Safety of Estrogens in Lupus Erythematosus National Assessment; SLEDAI: Systemic Lupus Erythematosus disease activity index; n: number; SLE: Systemic Lupus Erythematosus; IS: patients who were receiving an immune-suppressor drug at the time of vaccination. Non-IS: patients who were not receiving an immune-suppressor drug at the time of vaccination; M: median; IQR: interquartile range. In bold, we indicate the significant *p* values.

## Data Availability

The raw data generated and/or analyzed in the present study are available in our institutional repository (rawdata.inmi.it) (accessed on 11 March 2025), subject to registration. The data can be found by selecting the article of interest from a list of articles ordered by the year of publication. No charge for granting access to data is required. In the event of a malfunction of the application, the request can be sent directly by email to the library (biblioteca@inmi.it) (accessed on 11 March 2025).
